# Transcatheter edge-to-edge repair in symptomatic patients with severe tricuspid regurgitation and prior heart valve surgery

**DOI:** 10.1016/j.xjon.2026.101613

**Published:** 2026-02-03

**Authors:** Stephen H. McKellar, Brian K. Whisenant, Gagan D. Singh, Susheel Kodali, Samir Kapadia, Shamir R. Mehta, David M. Lasorda, Nadira Hamid, Vinod Thourani, Zexu Lin, Kelli Peterman, Rebecca T. Hahn, Gilbert H.L. Tang, Paul Sorajja, David H. Adams

**Affiliations:** a Intermountain Medical Center, Salt Lake City, Utah; b University of California Davis Medical Center, Sacramento, Calif; c Columbia University Irving Medical Center/New York-Presbyterian Hospital, New York, NY; d The Cleveland Clinic Foundation, Cleveland, Ohio; e Hamilton Health Science Center, Hamilton, Ontario, Canada; f Allegheny General Hospital, Pittsburgh, Pa; g Columbia University Irving Medical Center, New York, NY; h Piedmont Healthcare, Atlanta, Ga; i Abbott Structural Heart, Santa Clara, Calif; j New York-Presbyterian/Columbia University Medical Center, New York, NY; k Mount Sinai Hospital, New York, NY; l Minneapolis Heart Institute, Minneapolis, Minn

**Keywords:** tricuspid regurgitation, transcatheter edge-to-edge repair, heart valve surgery

## Abstract

**Background:**

Tricuspid valve surgery for tricuspid regurgitation (TR) following prior valve surgery carries increased risk. This study evaluated 2-year outcomes of tricuspid transcatheter edge-to-edge repair (T-TEER) in patients with severe TR and prior valve surgery.

**Methods:**

TRILUMINATE Pivotal is an international randomized trial comparing T-TEER with the TriClip (device group) to medical therapy (control group) in patients with symptomatic severe TR, with a concurrent single-arm cohort (device) for anatomically complex patients. Echocardiograms were assessed in a core laboratory, and outcomes were adjudicated by an independent clinical events committee.

**Results:**

Among the 469 patients in the device group, 113 had prior valve surgery and 356 did not. Baseline characteristics were comparable in the 2 groups: mean age, 77 years versus 79 years; female sex, 64% versus 59%; atrial fibrillation, 87% versus 86%; and heart failure hospitalization (HFH) within 1 year prior to T-TEER, 26% versus 25%. The T-TEER success rate was 99% in both groups, with no in-hospital deaths, a median hospital stay of 1 day, and 97% discharged home. Thirty-day adverse event rates were low and similar: rates of all-cause mortality, stroke, and new pacemaker implantation were all <2%, and the rate of major bleeding was <4%. At 2 years, outcomes remained favorable and comparable: ≤ moderate TR was achieved in 81% versus 80% (*P* = .93), New York Heart Association class I/II was observed in 74% versus 84% (*P* = .11), and KCCQ scores improved by a mean of 15 ± 21 points versus 16 ± 23 points (*P* = .66). Both groups experienced a significant reduction in HFH (79% vs 31%; *P* = .01).

**Conclusions:**

In patients with prior valve surgery, T-TEER was safe and resulted in significant TR reduction and symptom improvement.

**Figure:**
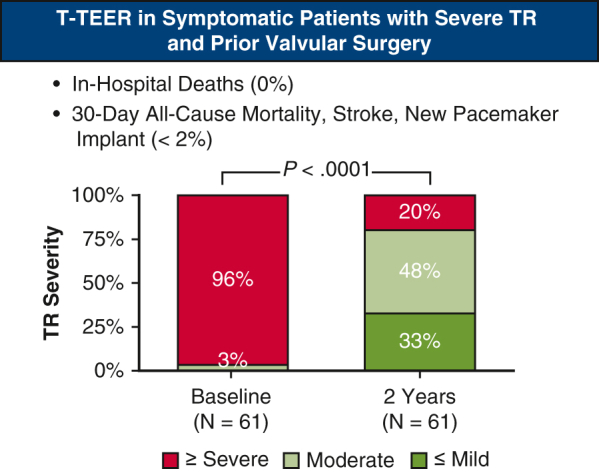
In patients with prior valve surgery, T-TEER was safe and resulted in significant TR reduction.

**Central Message**

Tricuspid transcatheter edge-to-edge repair is safe and effective in patients with severe tricuspid regurgitation (TR) and prior valve surgery, offering significant TR reduction and symptom improvement.

**Perspective**

Patients with severe tricuspid regurgitation (TR) and a history of prior valve surgery often face elevated surgical risks and limited treatment options. This study provides important insights that tricuspid transcatheter edge-to-edge repair offers a minimally invasive alternative with favorable safety and clinical outcomes, including significant TR reduction and improved functional status.

Severe tricuspid regurgitation (TR) often leads to substantial morbidity, mortality, and diminished quality of life.^1^ Patients typically present with symptoms of congestive right heart failure that contribute to poor long-term survival.^2^ Treatment options for severe TR traditionally have been limited to medical therapies and heart valve surgery. Medical management may provide symptom relief but does not treat the underlying disease directly.^3^ Surgery is not commonly performed in patients with isolated severe TR. Studies have shown an in-hospital mortality rate ranging from 8.8% to 16.8% for patients undergoing isolated tricuspid valve surgery after prior left-sided valve surgery or reoperative tricuspid valve surgery.^4^, ^5^, ^6^, ^7^, ^8^, ^9^ These findings highlight the need for safer therapeutic strategies for the treatment of TR, particularly in patients who would be candidates for reoperation.

Tricuspid transcatheter edge-to-edge repair (T-TEER) has emerged as a promising minimally invasive procedure for treating TR. Recent studies of T-TEER have demonstrated low complication rates, significant and durable reductions in TR severity, improved quality of life, and a decreased rate of heart failure hospitalization (HFH).^10^, ^11^, ^12^, ^13^, ^14^ The aim of this analysis was to evaluate the 2-year safety and clinical outcomes of T-TEER in patients with prior heart valve surgery.

## Methods

### Study Design and Analysis Population

The Trial to Evaluate Cardiovascular Outcomes in Patients Treated with the Tricuspid Valve Repair System Pivotal (TRILUMINATE Pivotal; NCT03904147) is an international, multicenter, prospective randomized controlled trial of T-TEER performed with the TriClip Transcatheter Tricuspid Valve Repair system (Abbott) or medical therapy in symptomatic patients with severe TR. The trial also included a concurrent, single-arm cohort of anatomically complex patients. The study protocol, patient eligibility criteria (Table E1), follow-up, and endpoints have been described in detail previously.^10^^,^^15^ In brief, patients with symptomatic severe or greater TR, verified by an echocardiographic core laboratory, with pulmonary artery systolic pressure <70 mm Hg, who had been on stable optimal medical therapy for at least 30 days, and were at intermediate or greater surgical risk (as evaluated by the local heart team) were eligible for enrollment. All patients provided written informed consent. Follow-up visits were required at 30 days, 6 months, 12 months, 18 months, and annually through 24, 36, 48, and 60 months. Results through 2 years are reported herein.

This study was conducted in accordance with the ethical principles outlined in the Declaration of Helsinki and was reviewed and approved by the Institutional Review Board at each participating site (2019-2021). Written informed consent for publication was obtained from all patients.

The study’s analysis population comprised patients with an attempted T-TEER procedure from the randomized and single-arm cohorts who had undergone prior heart valve surgery at least 60 days prior to screening for the TRILUMINATE Pivotal trial.

### Study Variables and Outcome Assessments

Clinical status was assessed using the New York Heart Association (NYHA) functional class and the Kansas City Cardiomyopathy Questionnaire (KCCQ) overall summary score. Echocardiographic assessment was performed according to current guidelines.^16^ TR severity was assessed using a 5-grade scale^17^: trace/mild, moderate, severe, massive, and torrential. All echocardiographic data, including TR severity, were assessed by the independent echocardiographic core laboratory. Major adverse events (MAEs) through 30 days, including cardiovascular mortality, endocarditis requiring surgery, new-onset renal failure, and nonelective cardiovascular surgery for device-related adverse events (AEs), were adjudicated by an independent clinical events committee, as were additional AEs through 2 years. New permanent pacemaker implantation was site-assessed.

### Statistical Analysis

Statistical analyses were conducted post hoc. Continuous variables are presented as mean ± standard deviation (SD), and categorical variables are presented as the percentage of patients with available data. The Bowker test was used to compare changes in TR severity and NYHA class across timepoints, and the Wilcoxon signed-rank test was used for KCCQ score comparisons to account for nonparametric data. Only patients with data recorded at all time points were included in the paired-analysis outcomes. The Kaplan-Meier method was used to estimate the survival rate and 95% confidence interval of all-cause mortality over 2 years. Confidence intervals and *P* values for annualized HFH rates were obtained using Poisson regression. Statistical significance was defined as a 2-sided *P* value < .05. All statistical analyses were performed using SAS version 9.4 (SAS Institute).

## Results

### Study Population and Baseline Characteristics

**Figure E1:**
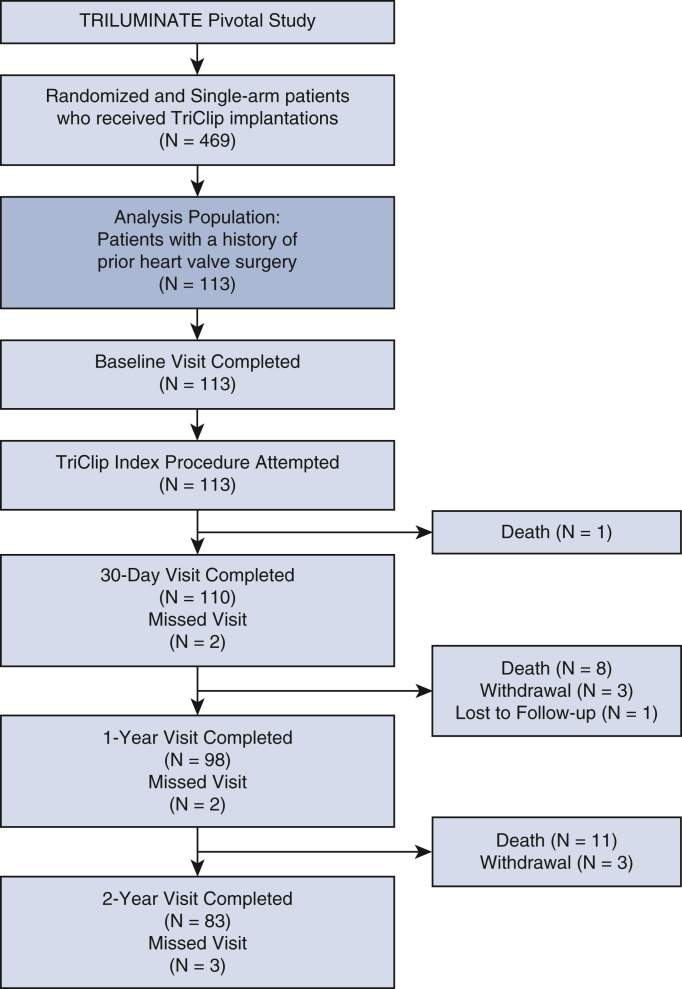
Study flowchart and patient disposition. Follow-up visits were calculated from the date of the index procedure. The 30-day visit window was defined as occurring between 27 days and 44 days postprocedure (-3 ± 14 days). The 1-year visit window was defined as occurring at 365 ± 28 days postprocedure. The 2-year visit window was defined as occurring at 730 ± 28 days postprocedure.

Between August 2019 and August 2022, a total of 469 patients from the randomized device group and the single-arm cohort underwent T-TEER, of whom 113 patients (65 from the randomized device group and 48 from the single-arm cohort) had a history of prior heart valve surgery (Table 1). The study flowchart and visit accountability are provided in Figure E1. Among the patients with prior heart valve surgery, 86.7% (n = 98) had prior surgery on 1 valve, and the remaining 13.3% (n = 15) had prior surgery on 2 valves. All but 3 patients (97.3%; n = 110) had a history of left-sided heart valve surgery, and 8.0% (n = 9) had prior tricuspid valve surgery. Among the 111 patients with recorded dates of prior heart valve surgery, the average (minimum-maximum) and median (interquartile range [IQR]) intervals between the most recent heart valve surgery and the T-TEER index procedure were 11 (0-53) years and 9 (5- 15) years, respectively. Most heart valve surgeries (81.1%) were performed between 2000 and 2020, and 8.1% were performed after 2020.

**Table 1:** Baseline characteristics of patients with and patients without prior heart valve surgery

Characteristic	Patients with prior heart valve surgery (N = 113)	Patients without prior heart valve surgery (N = 356)	*P* value
Demographics			
Age, y, mean ± SD	76.7 ± 8.0 (113)	79.2 ± 7.8	.0029
Age ≥75 y, % (n/N)	70.0 (79/113)	80.0 (284/356)	.0289
Female sex, % (n/N)	63.7 (72/113)	58.7 (209/356)	.3438
BMI, kg/m^2^, mean ± SD	26.6 ± 5.3 (113)	26.5 ± 5.5 (356)	.8645
Laboratory results, mean ± SD			
BNP, pg/mL	315.4 ± 272.7 (60)	424.9 ± 406.6 (194)	.0179
NT-proBNP, pg/mL	1516.9 ± 1193.7 (53)	2208.0 ± 1833.4 (147)	.0159
eGFR, mL/min/1.73 m^2^	54.9 ± 19.2 (112)	54.4 ± 20.9 (342)	.8077
Blood urea nitrogen, mg/dL	29.6 ± 15.7 (111)	30.5 ± 17.3 (338)	.6312
Hemoglobin, g/dL	12.2 ± 1.9 (112)	12.6 ± 1.9 (342)	.0497
Bilirubin, mg/dL	0.9 ± 0.5 (111)	1.0 ± 0.6 (341)	.1643
INR	1.9 ± 0.9 (95)	1.6 ± 0.7 (312)	.0008
MELD score	15.6 ± 6.1 (94)	14.3 ± 5.4 (308)	.0401
Serum creatinine, m/dL	1.3 ± 0.4 (112)	1.3 ± 0.5 (342)	.3058
Comorbidities, % (n/N)			
Myocardial ischemia	4.4 (5/113)	3.9 (14/356)	.8172
Liver disease	5.3 (6/113)	7.3 (26/356)	.4640
TIA	8.0 (9/113)	5.9 (21/356)	.4343
COPD	12.4 (14/113)	15.7 (56/356)	.3852
Stroke	13.3 (15/113)	6.2 (22/356)	.0148
Diabetes mellitus	15.9 (18/113)	17.4 (62/356)	.7144
Coronary artery bypass grafting	20.4 (23/113)	16.9 (60/356)	.3957
CIED	25.7 (29/113)	26.4 (94/356)	.8761
Renal disease	26.5 (30/113)	37.4 (133/356)	.0355
Dyslipidemia	58.4 (66/113)	64.0 (228/356)	.2803
Hypertension	76.1 (86/113)	82.6 (294/356)	.2803
Atrial fibrillation	86.7 (98/113)	85.7 (305/356)	.7794
HFH within 1 y before enrollment	25.7 (29/113)	25.3 (90/356)	.9351
Hemodynamic measurements, mean ± SD			
Systolic pulmonary artery pressure, mmHg	40.3 ± 10.2 (113)	38.0 ± 9.6 (356)	.0348
Mean pulmonary artery pressure, mmHg	25.3 ± 5.9 (113)	24.5 ± 6.1 (356)	.1811
Pulmonary capillary wedge pressure, mmHg	15.0 ± 4.6 (113)	14.3 ± 4.6 (356)	.1612
Central venous pressure, mmHg	11.4 ± 6.4 (59)	11.7 ± 4.9 (203)	.5414
TR severity, % (n/N)			.1549
Moderate	2.8 (3/106)	0.9 (3/349)	
Severe	23.6 (25/106)	19.2 (67/349)	
Massive	14.2 (15/106)	21.2 (74/349)	
Torrential	59.4 (63/106)	58.7 (205/349)	
TR etiology, % (n/N)			.4012
Degenerative	3.6 (4/111)	2.6 (9/352)	
Functional	93.7 (104/111)	91.2 (321/352)	
Mixed	1.8 (2/111)	2.3 (8/352)	
Pacer-related	0.9 (1/111)	4.0 (14/352)	
Echocardiographic measurements, mean ± SD (N)			
RVEDD mid (4Ch), cm	3.7 ± 0.8 (109)	3.8 ± 0.8 (349)	.4252
RVEDD base (4Ch), cm	5.1 ± 0.9 (111)	5.1 ± 0.9 (349)	.8319
Tricuspid annulus diameter, cm	4.4 ± 0.9 (111)	4.4 ± 0.7 (350)	.3669
RV TAPSE, cm	1.6 ± 0.4 (111)	1.7 ± 0.4 (348)	.0044
RA volume, mL	153.0 ± 99.4 (111)	156.8 ± 88.5 (349)	.718
LVEF, %	58.3 ± 9.6 (103)	59.9 ± 9.0 (338)	.1175
Clinical presentation			
KCCQ overall score, mean ± SD (N)	53.5 ± 20.9 (112)	54.6 ± 23.2 (356)	.6515
6MWD, m, mean ± SD (N)	250.1 ± 122.0 (106)	230.3 ± 118.4 (340)	.1373
NYHA functional class III/IV, % (n/N)	61.1 (69/113)	56.5 (201/356)	.3886

Prior heart valve surgery, % (n/N)	Patients with prior heart valve surgery (N = 113)
Single heart valve surgery	86.7 (98/113)
Double heart valve surgery	13.3 (15/113)
Left-sided heart valve surgery	97.3 (110/113)
Time between T-TEER index procedure and most recent heart valve surgery, y	
Mean ± SD	11 ± 9 (111)
Median (IQR)	9 (5-15)
Range (minimum-maximum)	(0-53)
Year of most recent heart valve surgery, % (n/N)	
2020 or later	8.1 (9/111)
2000-2020	81.1 (90/111)
1980-2000	9.9 (11/111)
Before 1980	0.9 (1/111)
Aortic valve only, % (n/N)	28.3 (32/113)
Replacement	23.9 (27/113)
Repair	3.5 (4/113)
Other	0.9 (1/113)
Pulmonic valve only, % (n/N)	0.0 (0/113)
Tricuspid valve only, % (n/N)	2.7 (3/113)
Repair	0.9 (1/113)
Other	1.8 (2/113)
Mitral valve only, % (n/N)	55.8 (63/113)
Replacement	19.5 (22/113)
Repair	35.4 (40/113)
Other	0.9 (1/113)
Tricuspid and mitral valves, % (n/N)	4.4 (5/113)
Tricuspid repair and mitral replacement	2.7 (3/113)
Both repair	1.8 (2/113)
Aortic and pulmonic valves, % (n/N)	
Both replacement	0.9 (1/113)
Aortic and tricuspid valves, % (n/N)	
Aortic replacement and tricuspid repair	0.9 (1/113)
Aortic and mitral valves, % (n/N)	7.1 (8/113)
Both replacement	4.4 (5/113)
Aortic replacement and mitral repair	1.8 (2/113)
Both repair	0.9 (1/113)

*SD*, Standard deviation; *BMI*, body mass index; *BNP*, B-type natriuretic peptide; *NT-proBNP*, N-terminal pro B-type natriuretic peptide; *eGFR*, estimated glomerular filtration rate; *INR*, international normalized ratio; *MELD*, model for end-stage liver disease; *TIA*, transient ischemic attack; *COPD*, chronic obstructive pulmonary disease; *CIED*, cardiac implantable electronic device; *HFH*, heart failure hospitalization; *TR*, tricuspid regurgitation; *RVEDD*, right ventricular end-diastolic dimension; *4Ch*, 4-chamber view; *RV*, right ventricular; *TAPSE*, tricuspid annular plane systolic excursion; *RA*, right atrium; *LVEF*, left ventricular ejection fraction; *KCCQ*, Kansas City Cardiomyopathy Questionnaire; *6MWD*, 6-minute walk distance; *NYHA*, New York Heart Association; *T-TEER*, tricuspid transcatheter edge-to-edge repair; *IQR*, interquartile range.

The patients’ baseline characteristics are provided in Table 1. The mean age was 76.7 ± 8.0 years, and 53.7% were women. The mean MELD (Model for End-Stage Liver Disease) score was 15.6. Patients exhibited elevated levels of B-type natriuretic peptide (315.4 ± 272.7 pg/mL) and N-terminal pro B-type natriuretic peptide (1516.9 ± 1193.7 pg/mL) and a low estimated glomerular filtration rate (54.9 ± 19.2 mL/min/1.73 m^2^), suggesting impaired cardiac and kidney function, presumably from venous hypertension. Other common comorbidities included atrial fibrillation in 86.7% of patients, hypertension in 76.1%, dyslipidemia in 58.4%, and renal disease in 26.5%. Many patients had a history of coronary artery bypass graft (20.4%), an existing cardiac implantable electronic device (CIED; 25.7%), and HFH within 1 year prior to enrollment (25.7%). Systolic and mean pulmonary artery pressures (40.3 ± 10.2 mmHg and 25.3 ± 5.9 mmHg, respectively), as well as pulmonary capillary wedge pressures (15.0 ± 4.6 mmHg), were mildly elevated. More than one-half of the patients had torrential TR (59.4%). TR etiology was predominantly functional (93.7%), followed by degenerative (3.6%), mixed (1.8%), and CIED lead-related (0.9%). Patients exhibited signs of adverse right heart remodeling, characterized by a dilated right ventricle (mean, 5.1± 0.9 cm, base), a dilated tricuspid valve annulus (4.4 ± 0.9 cm), and an enlarged right atrium (153.0 ± 99.4 mL). Right ventricular dysfunction (tricuspid annular plane systolic excursion <1.7 cm) and left ventricular dysfunction (left ventricular ejection fraction <50%) were observed in 62.2% and 15.5% of patients, respectively. The overall baseline KCCQ score (53.5 ± 20.9) indicated a moderate impairment in quality of life. A majority of patients (61.1%) presented with severe heart failure symptoms at baseline, as indicated by NYHA functional class III/IV.

### T-TEER Procedure Outcomes

Procedural outcomes are presented in Table 2. The device was implanted successfully in 112 of the 113 patients (99.1%), with an average of 2.1 ± 0.8 clips per patient. There were no in-hospital deaths. In the single unsuccessful case, the device could not be implanted owing to an inability to adequately grasp the leaflets. The patient did not undergo a second implantation attempt and withdrew from the study with no AEs reported. Most patients received 2 clips (55.8%), 20.4% received 1 clip, 18.6% had 3 clips, and 4.4% had 4 clips. The total procedure time and device time were 152.8 ± 74.9 minutes and 88.1 ± 65.8 minutes, respectively. The median hospital length of stay was 1.0 (IQR, 1.0-2.0) days, and 97.3% of the patients were discharged to home.

**Table 2:** Procedural outcomes of patients with and patients without prior heart valve surgery

Outcome	Patients with prior heart valve surgery (N = 113)	Patients without prior heart valve surgery (N = 356)	*P* value
Successful implantation, % (n/N)	99.1 (112/113)	98.6 (351/356)	.6685
Number of clips implanted			.1332
Mean ± SD	2.1 ± 0.8 (113)	2.1 ± 0.7 (356)	
Median (IQR)	2 (2-2)	2 (2-3)	
Number of clips implanted, % (n/N)			.1124
0	0.9 (1/113)	1.4 (5/356)	
1	20.4 (23/113)	12.9 (46/356)	
2	55.8 (63/113)	57.6 (205/356)	
3	18.6 (21/113)	26.1 (93/356)	
4	4.4 (5/113)	2.0 (7/356)	
TriClip generation, % (n/N)			.8424
TriClip	31.9 (36/113)	32.9 (117/356)	
TriClip G4	68.1 (77/113)	67.1 (239/356)	
Total procedure time, min∗			.9023
Mean ± SD	152.8 ± 74.9 (112)	150.6 ± 69.5 (354)	
Median (IQR)	136.0 (96.5-186.5)	139.5 (100.0-188.0)	
Device time, min†			.7321
Mean ± SD	88.1 ± 65.8 (112)	87.2 ± 61.0 (350)	
Median (IQR)	68.0 (43.0-111.5)	73.00 (46.0-112.0)	
Hospital length of stay, d			.0904
Mean ± SD	1.5 ± 1.0 (113)	1.7 ± 2.1 (356)	
Median (IQR)	1.0 (1.0-2.0)	1.0 (1.0-2.0)	
ICU stay, % (n/N)d			.0015
ICU length of stay, d	5.3 (6/113)	10.7 (38/356)	
Mean ± SD	1.0 ± 0.0 (6)	1.8 ± 1.5 (38)	
Median (IQR)	1.0 (1.0-1.0)	1.0 (1.0-2.0)	
Discharge status, % (n/N)			.7156
Home	97.3 (110/113)	96.6 (344/356)	
Nursing home	1.8 (2/113)	1.4 (5/356)	
Rehabilitation center	0.9 (1/113)	2.0 (7/356)	
In-hospital mortality, % (n/N)	0.0 (0/113)	0.0 (0/356)	-

*SD*, Standard deviation; *IQR*, interquartile range; *ICU*, intensive care unit.

∗ Total procedure time was defined as the time between the earliest insertion of either the TEE probe or steerable guide catheter and the removal of the last catheter and TEE probe.

† Device time was defined as the time between insertion of the steerable guide catheter and retraction of the TriClip delivery system into the steerable guide catheter.

### Safety Outcomes

Freedom from MAEs through 30 days was achieved in 99.1% of the patients. One patient, an 85-year-old woman with multiple preexisting conditions, including prior mitral valve repair surgery, hypertension, dyslipidemia, and a CIED, experienced cardiac arrest at 11 days postprocedure (Table 3). This cardiovascular death was adjudicated as non–heart failure-related and unrelated to the device and procedure.

**Table 3:** Safety profile of T-TEER in patients with and patients without prior heart valve surgery

Safety profile	Patients with prior heart valve surgery (N = 113)	Patients without prior heart valve surgery (N = 356)	*P* value
MAEs through 30 d, % (n/N)			
Any MAE	0.9 (1/113)	0.8 (3/356)	>.99
Cardiovascular mortality	0.9 (1/113)	0.3 (1/356)	
Endocarditis requiring surgery	0.0 (0/113)	0.0 (0/356)	
New-onset renal failure	0.0 (0/113)	0.6 (2/356)	
Nonelective CV surgery for device-related AEs	0.0 (0/113)	0.0 (0/356)	

	Through 30 d	Through 1 y	Through 2 y	Through 30 d	Through 1 y	Through 2 y	
All-cause mortality, % (n/N)	0.9 (1/113)	8.0 (9/113)	17.7 (20/113)	0.3 (1/356)	11.8 (42/356)	19.4 (69/356)	.6910
Cardiovascular mortality	0.9 (1/113)	7.1 (8/113)	15.0 (17/113)	0.3 (1/356)	7.9 (28/356)	12.6 (45/356)	.5110
Heart failure-related	0.0 (0/113)	4.4 (5/113)	8.8 (10/113)	0.3 (1/356)	6.5 (23/356)	9.8 (35/356)	.7575
Non–heart failure-related	0.9 (1/113)	2.7 (3/113)	6.2 (7/113)	0.0 (0/356)	1.4 (5/356)	2.8 (10/356)	.1428
Noncardiovascular mortality	0.0 (0/113)	0.9 (1/113)	2.7 (3/113)	0.0 (0/356)	3.9 (14/356)	6.5 (23/356)	.1581
Hospitalization, % (n/N)	9.7 (11/113)	44.2 (50/113)	61.9 (70/113)	11.5 (41/356)	42.7 (152/356)	60.1 (214/356)	.7281
Heart failure hospitalization	1.8 (2/113)	14.2 (16/113)	16.8 (19/113)	2.2 (8/356)	16.9 (60/356)	26.4 (94/356)	.0378
Other cardiovascular hospitalization	3.5 (4/113)	13.3 (15/113)	20.4 (23/113)	1.7 (6/356)	9.6 (34/356)	17.4 (62/356)	.4799
Noncardiovascular hospitalization	4.4 (5/113)	23.9 (27/113)	37.2 (42/113)	5.9 (21/356)	25.3 (90/356)	37.6 (134)	.9280
Other AEs, % (n/N)							
Tricuspid valve surgery	0.0 (0/113)	2.7 (3/113)	4.4 (5/113)	0.3 (1/356)	1.7 (6/356)	2.2 (8/356)	.3196
Tricuspid valve reintervention	0.9 (1/113)	3.5 (4/113)	3.5 (4/113)	1.1 (4/356)	3.1 (11/356)	4.8 (17/356)	.7947
Major bleeding∗	2.7 (3/113)	†	†	3.7 (13/356)	†	†	.7716
New-onset renal failure	0.0 (0/113)	†	†	0.6 (2/356)	†	†	>.99
TIA	0.0 (0/113)	0.9 (1/113)	1.8 (2/113)	0.0 (0/356)	0.6 (2/356)	0.8 (3/356)	.5983
Stroke (VARC II)	0.0 (0/113)	0.9 (1/113)	2.7 (3/113)	0.3 (1/356)	0.6 (2/356)	1.1 (4/356)	.3669
Myocardial infarction	0.0 (0/113)	†	†	0.0 (0/356)	†	†	-
Nonelective CV surgery for device-related AEs	0.0 (0/113)	†	†	0.0 (0/356)	†	†	-
Cardiogenic shock	0.0 (0/113)	0.0 (0/113)	0.0 (0/113)	0.3 (1/356)	0.3 (1/356)	0.6 (2/356)	>.99
SLDA‡	5.3 (6/113)	6.2 (7/113)§	6.2 (7/113)	7.6 (27/356)	7.6 (27/356)	7.9 (28/356)	.5560
Device embolization‡	0.0 (0/113)	0.0 (0/113)	0.0 (0/113)	0.0 (0/356)	0.0 (0/356)	0.0 (0/356)	-
Device thrombosis‡	0.0 (0/113)	0.0 (0/113)	0.0 (0/113)	0.0 (0/356)	0.0 (0/356)	0.0 (0/356)	-
New permanent pacemaker implantation‖	1.8 (2/113)	3.5 (4/113)	5.3 (6/113)	0.3 (1/356)	3.4 (12/356)	5.1 (18/356)	.9151

MAEs and safety profile AEs were counted from the index procedure date. For MAEs, *P* values were calculated for 30-day event rate comparisons. For the safety profile, *P* values were calculated for 2-year event rate comparisons, except for AEs adjudicated through 30 days, for which 30-day comparisons were applied.

*MAE*, Major adverse event; *CV*, cardiovascular; *AE*, adverse event; *TIA*, transient ischemic attack; *VARC*, Valve Academic Research Consortium; *SLDA*, single leaflet device attachment.

∗ Major bleeding was defined as bleeding type ≥3 based on a modified Bleeding Academic Research Consortium definition.

† These AEs were adjudicated through 30 days.

‡ These AEs were evaluated by the echocardiographic core laboratory and were calculated from the index procedure date through the upper bound of the 30-day visit window (ie, 44 days), 1-year visit window (ie, 393 days), and 2-year visit window (ie, 758 days).

§ One patient with an SLDA had a delayed 30-day visit (113 days after the index procedure), and thus this SLDA event was included in the “through 1 year and “through 2 years windows.

‖ New pacemaker implantation was site-assessed.

Safety profiles and specific AEs through 30 days and 2 years postprocedure are reported in Table 3. Within 30 days, there were no reports of tricuspid valve surgery, new-onset renal failure, transient ischemic attack, stroke, myocardial infarction, cardiogenic shock, device embolization, or device thrombosis. The single non–heart failure-related death reported through 30 days corresponds to the case in the MAEs. Other 30-day AEs, including tricuspid valve reintervention (0.9%), HFH (1.8%), major bleeding (2.7%), new permanent pacemaker implantation (1.8%), and single leaflet device attachment (5.3%), were infrequent. At 2 years, AE rates remained low, including rates of new permanent pacemaker implantation (5.3%), tricuspid valve surgery (4.4%), tricuspid valve reintervention (3.5%), stroke (2.7%), cardiovascular mortality (15.0%), and HFH (16.8%). No new occurrences of device embolization and device thrombosis were reported at the 30-day follow-up.

### Changes in TR Severity and Echocardiographic Evaluation of the Right Heart

**Figure 1:**
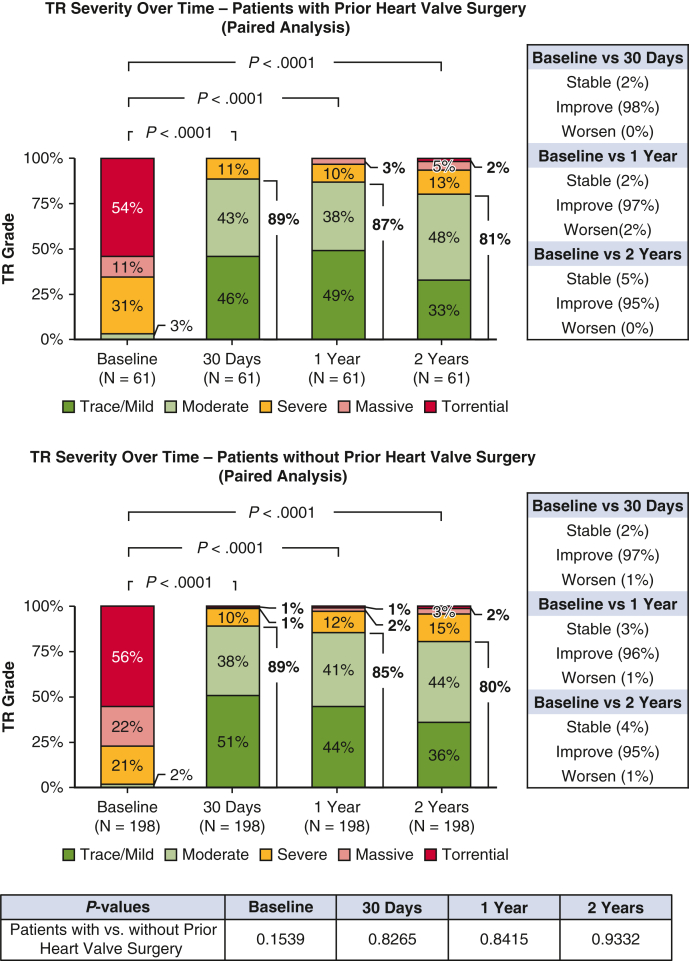
Severity of TR at baseline, 30 days, 1 year, and 2 years (paired analysis). In this analysis, any follow-up data collected after patients underwent tricuspid valve surgery during the follow-up period were censored.

The paired analysis of changes in TR severity through the 2-year follow-up is illustrated in Figure 1. Almost all patients (95%) experienced at least a 1-grade reduction in TR severity at 2 years compared to baseline, and no patients had worsening TR severity. TR severity was reduced significantly, with 81% of patients having moderate or less TR at 2 years, compared to 3% at baseline (*P* < .0001). Small changes in cardiac remodeling were observed at 2 years (Table E2).

### Functional and Clinical Outcomes

**Figure 2:**
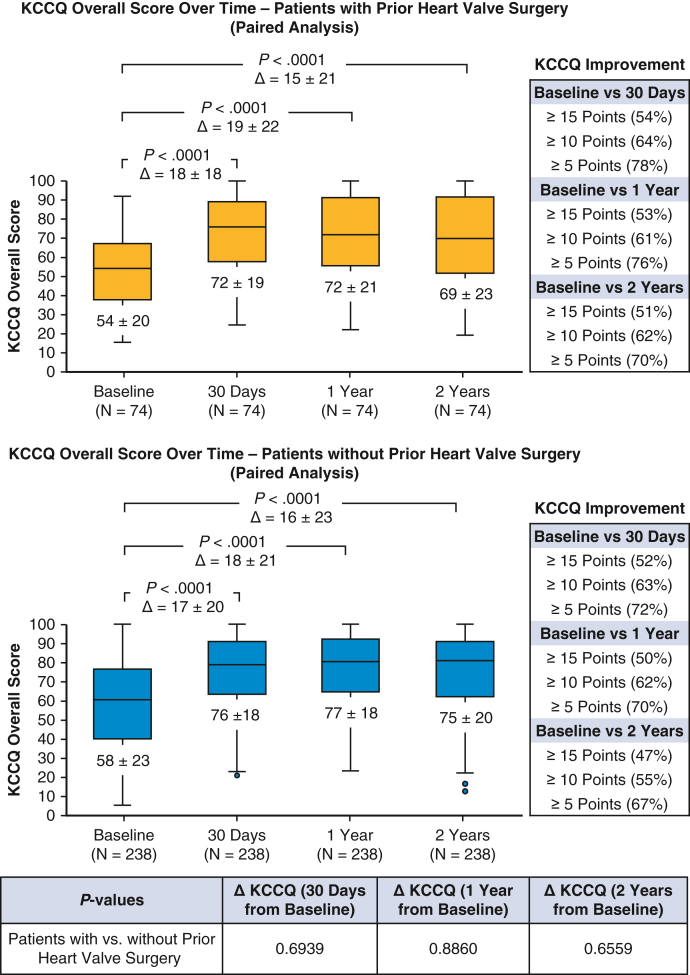
Kansas City Cardiomyopathy Questionnaire overall score at baseline, 30 days, 1 year, and 2 years (paired analysis). The lower and upper borders of the box represent the 25th and 75th percentiles. The *middle horizontal line* represents the median. The lower and upper whiskers represent the minimum and maximum values of nonoutliers. Extra *dots* represent outliers. The mean ± SD value is shown beneath the lower borders of the box. In this analysis, any follow-up data collected after patients underwent tricuspid valve surgery during the follow-up period were censored.

**Figure 3:**
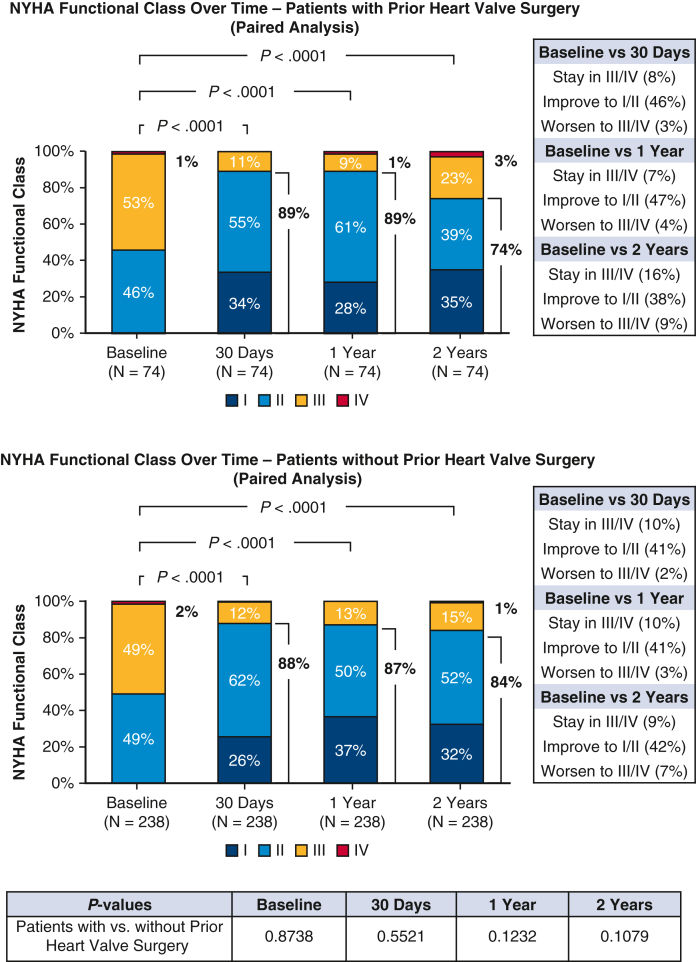
New York Heart Association functional class at baseline, 30 days, 1 year, and 2 years (paired analysis). In this analysis, any follow-up data collected after patients underwent tricuspid valve surgery during the follow-up period were censored.

**Figure E2:**
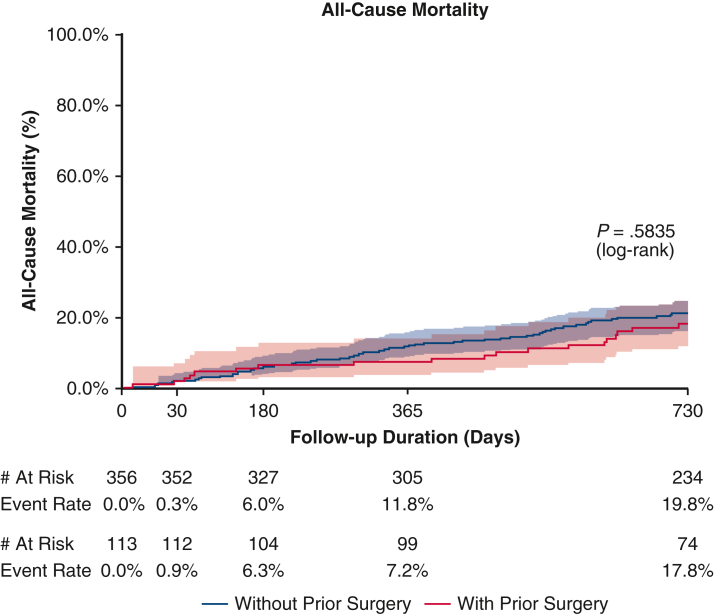
Kaplan-Meier curve for all-cause mortality through 730 days from the index procedure. The shaded are indicates 95% confidence interval. Note that in this analysis, any follow-up data collected after patients underwent tricuspid valve surgery during the follow-up period were censored.

**Figure E3:**
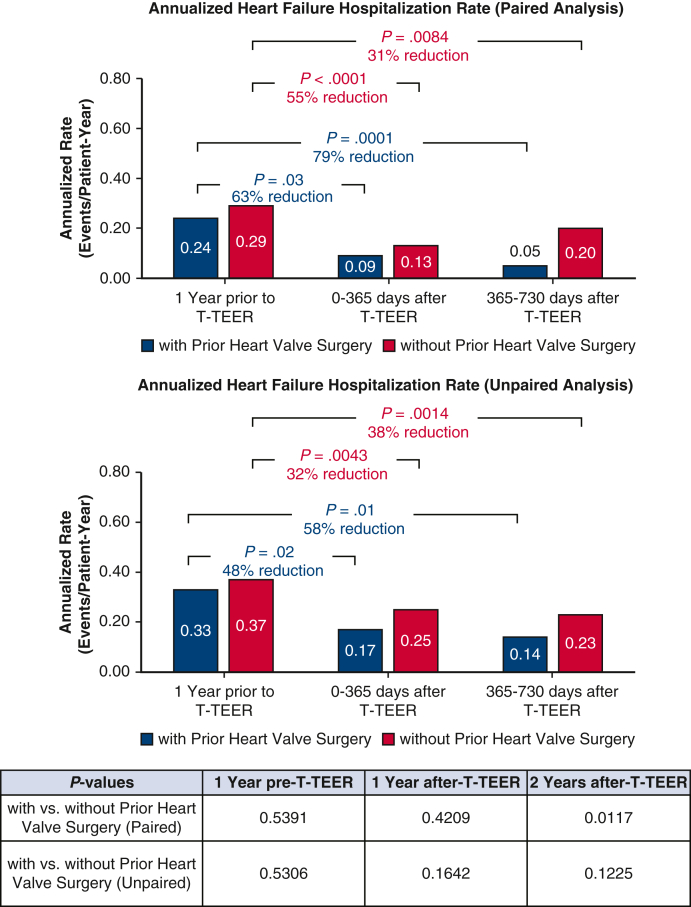
Annualized heart failure hospitalization rate. All patients were considered for the unpaired analysis; any follow-up data collected after patients underwent tricuspid valve surgery during the follow-up period were censored. For the paired analysis, individuals who either did not survive the 2-year follow-up or underwent tricuspid valve surgery within that interval were excluded. *T-TEER*, Tricuspid transcatheter edge-to-edge repair.

The paired analysis of KCCQ overall score (Figure 2) indicated a substantial improvement of 18 ± 18 points from baseline (54 ± 20 points) to 30 days (72 ± 19 points; *P* < .0001). This significant improvement was sustained at both 1 year and 2 years (*P* < .0001 for each comparison with baseline). At 2 years, more than one-half (51%) of the patients experienced a large KCCQ improvement (defined as ≥ 15-point improvement) compared with baseline. The proportion of patients classified as NYHA class I/II increased significantly, from 46% at baseline to 74% at 2 years (*P* < .0001) (Figure 3). The Kaplan-Meier estimates of all-cause mortality was 7.2% at 1 year and 17.8% at 2 years postprocedure (Figure E2). When assessing HFH during follow-up, the annualized rate decreased from 0.24 events per patient-year at 1 year preprocedure to 0.05 events per patient-year at 2 years (ie, 365-730 days) postprocedure, a significant reduction of 79% (*P* = .0001; Figure E3).

### Comparison With Patients Without Prior Heart Valve Surgery

Baseline characteristics were generally comparable between patients with and patients without prior heart valve surgery (Table 1), with minor variations observed in the proportion of patients age ≥75 years (70.0% vs 80.0%; *P* = .0289), history of stroke (13.3% vs 6.2%; *P* = .0148), and renal disease (26.5% vs 37.4%; *P* = .0355). Procedural success (∼99%), clip usage (2 clips on average), and acute outcomes (median in-hospital stay, ∼1.5 days; discharge to home, ∼97%; in-hospital death, 0%) were comparable in the 2 groups (Table 2). Rates of MAEs (<1.0%) and other AEs (Table 3) through 30 days were low and similar to those observed in patients without prior heart valve surgery. Over the 2-year follow-up period, the overall AE rates remained comparable in the 2 groups. TR reduction (moderate or less TR at 2 years, 81% vs 80%; *P* = .9332; Figure 1), quality of life improvements (ΔKCCQ score from baseline to 2 years, 15 ± 21 points vs 16 ± 23 points; *P* = .6559, Figure 2), symptom relief (NYHA functional class I/II at 2 years, 74% vs 84%; *P* = .1079; Figure 3) were also similar for patients with and those without prior heart valve surgery. Both groups experienced a significant reduction in HFH at 2 years following T-TEER compared with the previous year, with a greater magnitude of reduction seen in patients with prior heart valve surgery (79% vs 31%; *P* = .0117; Figure E3)

## Discussion

This analysis evaluated the acute and 2-year safety and clinical outcomes of T-TEER in patients presenting with symptomatic, severe TR and prior heart valve surgery. Despite the intermediate or greater estimated risk for mortality or morbidity in this patient cohort for isolated tricuspid valve surgery, our results indicate that T-TEER is a viable and safe therapeutic option. T-TEER in this population resulted in no in-hospital deaths, a 30-day MAE-free rate of 99.1%, and no occurrences of new tricuspid valve surgery, new-onset renal failure, myocardial infarction, or device embolization or thrombosis within 30 days of T-TEER. Furthermore, significant clinical improvements were observed alongside this excellent safety profile, evidenced by the sustained reduction in TR severity, durable improvements in KCCQ score, and increased proportion of patients classified as NYHA class I/II at the 2-year follow-up.

In clinical practice, patients with isolated severe TR often are not referred initially for surgical intervention until the disease has significantly progressed and become refractory to medical treatment. The reluctance to operate on patients who develop severe TR could stem from the lack of guideline recommendations^18^^,^^19^ and the high risk of operative death, especially in the context of heart valve reoperation. Numerous isolated tricuspid valve surgery studies^4^, ^5^, ^6^, ^7^, ^8^ (Table 4) of patients with severe TR and prior heart valve surgery have documented high in-hospital mortality rates, ranging from approximately 9% to 16%. Compared with the aforementioned studies, our analysis population had a notable comorbidity burden, older age, and a higher incidence of atrial fibrillation; however, our results demonstrate an excellent safety profile, as evidenced by no in-hospital mortality and remarkable 30-day (99.1%) and 1-year (92.8%) survival rates. These results indicate the potential of T-TEER as a therapeutic option for this patient population and may advocate for earlier intervention before the disease progresses to advanced right heart failure.

**Table 4:** Comparison of baseline characteristics and mortality outcomes: current study versus published data

Characteristic/outcome	T-TEER in patients with prior heart valve surgery	Isolated tricuspid valve surgery in patients with prior heart valve surgery				
	Current study (N = 113)∗	Staab et al^4^ (N = 34)†	Pfannmüller et al^8^ (N = 82)∗	Kim et al^5^ (N = 61)∗	Chen et al^6^ (N = 107)∗	Dreyfus et al^7^ ((N = 101)∗
Study design	Prospective, multicenter	Retrospective, single center	Retrospective, single center	Prospective, single center	Retrospective, single center	Retrospective, multicenter
Tricuspid valve surgery, %						
Repair	Not applicable	20.6	75.6	3.1	0.0	24.8
Replacement	Not applicable	79.4	24.4	86.9	100.0	75.2
History of HV surgery, %						
Prior HV surgery	100.0	100.0	-	-	88.8	100.0
Prior TV surgery	8.0	21.0	24.4	86.9	37.4	-
Prior left-sided surgery	97.3	100.0	72.5	93.4	86.0	100.0
Baseline characteristics						
Demographics						
Age, y, mean ± SD	76.7 ± 8.0	62.8 ± 12.0	64.1 ± 11.9	57.0 ± 8.9	53.6 ± 12.5	66 ± 11
Female sex, %	63.7	82.4	72.0	88.5	64.5	63.4
BMI, kg/m^2^, mean ± SD	26.6 ± 5.3	-	25.2 ± 4.2	-	22.6 ± 3.7	26 ± 5
Comorbidities, %						
Stroke	13.3	-	-	-	-	14.8
Diabetes mellitus	15.9	-	19.5	-	5.6	18.8
CIED	25.7	14.7	41.4	-	-	25.7
Hypertension	76.1	-	-	-	-	46.5
Atrial fibrillation	86.7	-	35.4	82.0	63.6	65.3
Hemodynamic measurements						
SPAP, mm Hg, mean ± SD	40.3 ± 10.2	44.2 ± 11.9	43.8 ± 21.8	41.5 ± 8.7	42.7 ± 10.3	43 ± 10
Echocardiography						
LVEF, %, mean ± SD	58.3 ± 9.6	49.7 ± 14.7	50.7 ± 23.5	57.4 ± 9.2	51.6 ± 6.2	55 ± 9
TR						
TR etiology, %	94 functional	41 organic	-	84 functional	-	100 functional
TR severity, %	97 severe or greater	-	92 TR 3+ to 4+	-	81.5 severe	-
Clinical presentation						
NYHA functional class III/IV, %	61.1	100.0	-	65.6	75.7	61.4
Mortality, %						
In-hospital mortality	0.0	8.8	-	9.8	16.8	15.8
30-d survival rate	99.1	89	85.4	-	-	85
1-y survival rate	92.8	75	-	-	-	78

The symbol “-” indicates that data were unavailable in the referenced publication.

*HV*, Heart valve; *TV*, tricuspid valve; *BMI*, body mass index; *CIED*, cardiac implantable electronic device; *SPAP*, systolic pulmonary artery pressure; *LVEF*, left ventricular ejection fraction; *TR*, tricuspid regurgitation; *NYHA*, New York Heart Association.

∗ Values are presented as mean ± standard deviation or %.

† Values are presented as mean ± standard error or %.

Patients undergoing elective tricuspid valve reoperation have significantly higher survival rates at both 30 days (96.0% vs 68.8%; *P* = .01) and 2 years (78.5% vs 38.4%; *P* < .001) compared to those undergoing nonelective (ie, urgent) procedures.^8^ In the context of the TRILUMINATE Pivotal study design, the T-TEER procedure in our analysis population could be considered elective. More data are needed to evaluate the safety outcomes of T-TEER in elective versus nonelective settings.

Echocardiographic guidance is critical in T-TEER procedures. However, acoustic shadowing and reverberation from previously implanted cardiac prostheses, such as prosthetic valves or annuloplasty devices, can present additional challenges in obtaining adequate imaging. Despite these potential obstacles, our data show that the success rate, safety, and efficacy of T-TEER in patients with prior heart valve surgery are not necessarily compromised by the presence of these prostheses. These findings suggest that with careful planning, T-TEER can be performed effectively even in patients with prior heart valve surgeries.

When comparing outcomes of T-TEER in patients with and those without prior cardiac surgery, our findings demonstrate comparable procedural success rates, similar safety profiles, and similar clinical benefits in both groups. Overall, these results further underscore the feasibility, safety, and therapeutic efficacy of T-TEER even in reoperative patients with symptomatic, severe TR. Considering the ongoing debates about optimal timing for surgical intervention in TR,^20^^,^^21^ the prognostic impact of T-TEER timing similarly warrants further investigation. Extended follow-up is recommended to comprehensively capture patient survival profiles and to evaluate the long-term efficacy of T-TEER in counteracting the progressive, negative impacts of TR. Future studies evaluating the learning curve and exploring potential outcome-volume relationships may offer valuable insights into procedural optimization and the impact of operator experience over time. Additionally, with the rapid and active advancement of tricuspid valve repair technologies, comparative studies evaluating clinical outcomes across different devices would be of interest as well. Such investigations could be beneficial for informing device selection and improving patient care in this evolving field.

### Study Limitations

First, the patients in this study were enrolled based on specific eligibility criteria, which may limit the generalizability of the observed outcomes to broader patient populations. Specifically, the study included mostly patients with functional or secondary TR, with few patients with degenerative or pacemaker lead-induced TR. This limitation is inherent to all prespecified clinical trials compared to real-world, all-comers studies, however. Second, the lack of randomization or stratification by prior valve surgery status may have introduced confounding factors. Although the 2 groups were generally comparable at baseline, the study did not use multivariable adjustment or matching techniques to assess comparability, owing primarily to sample size constraints. As such, the analysis was exploratory and intended for descriptive purposes, and the results should be interpreted with appropriate caution. Third, precise characterization of surgical risk in tricuspid valve interventions remains challenging. With the continuing development of more dedicated surgical risk calculators, future studies may be better positioned to compare the safety and efficacy of T-TEER and surgical treatment in high-risk patient populations. Although limitations exist, we believe that the outcomes reported in the present study are highly relevant for patients with functional TR and should encourage further research to aid clinical decision making. Additionally, the TRILUMINATE Pivotal trial is ongoing, and further outcomes will be evaluated as longer-term follow-up data become available.

## Conclusion

Patients with prior heart valve surgery could be considered at higher risk for surgical treatment of TR due to the operative risk associated with isolated tricuspid valve surgery. In this work, our data indicate that T-TEER using the TriClip system is a safe and effective treatment for severe TR in patients with prior heart valve surgery. Our findings support the use of T-TEER as a viable and safe alternative to surgical intervention, offering significant improvements in TR severity and quality of life with a favorable safety profile.

## Conflict of Interest Statement

Dr Whisenant reported receiving consulting fees from Abbott and Edwards Lifesciences. Dr Singh reported serving as a consultant and/or on advisory board member for Abbott Structural Heart, Boston Scientific, Siemens Healthcare, and Phillips. Dr Kodali reported receiving institutional research grants from Edwards Lifesciences, Medtronic, and Abbott; consulting fees from Abbott, Anteris Technologies, and Meril Lifesciences; and equity options from Biotrace Medical and Thubrikar Aortic Valve. Dr Mehta reported grant support from Abbott; personal fees from Amgen, Bristol Myers Squibb, Novartis, NovoNordisk, and Janssen; and board participation for Merck. Dr Lasorda reported consulting fees from Cardiovascular Systems, Edwards Lifesciences, and Shockwave Medical. Dr Hamid reported consulting fees from 4C Medical Technologies, Alleviant Medical, AMX, Anteris Technologies, Edwards Lifesciences, Philips, Valcare Med, Vdyne, and WL Gore & Associates. Dr Thourani reported receiving research grants from and serving as an advisor for Edwards Lifesciences. Dr Lin and Ms Peterman are salaried employees of Abbott. Dr Hahn reported speaking fees from Abbott Structural, Baylis Medical, Edwards Lifesciences, and Philips Healthcare; institutional consulting contracts (for which she receives no direct compensation) with Abbott Structural, Anteris, Boston Scientific, Edwards Lifesciences, Medtronic, Novartis, and Philips Healthcare; serving as Chief Scientific Officer for the Echocardiography Core Laboratory at the Cardiovascular Research Foundation for multiple industry-sponsored valve trials (for which she receives no direct industry compensation). Dr Tang reported receiving speaker's honoraria and serving as a physician proctor, consultant, advisory board member, TAVR publications committee member, RESTORE study steering committee member, APOLLO trial screening committee member, and IMPACT MR steering committee member for Medtronic; receiving speaker’s honoraria and serving as a physician proctor, consultant, advisory board member, and TRILUMINATE trial anatomic eligibility and publications committee member for Abbott Structural Heart; serving as an advisory board member for Boston Scientific and JenaValve, as a consultant and physician screening committee member for Shockwave Medical, and consultant for NeoChord, Peija Medical, and Shenqi Medical Technology; and receiving speaker's honoraria from Siemens Healthineers. Dr Sorajja is the co-principal investigator for the TRILUMINATE Pivotal trial and reported serving on the advisory board for Anteris and VDyne and receiving consulting fees from Boston Scientific, Edwards Lifesciences, Evolution Medical, Medtronic, Shifamed, TriFlo, and WL Gore & Associates. Dr Adams is the co-principal investigator for the TRILUMINATE Pivotal trial and reported receiving royalties from Edward Lifesciences and Medtronic. All other authors reported no conflicts of interest.

The *Journal* policy requires editors and reviewers to disclose conflicts of interest and to decline handling or reviewing manuscripts for which they may have a conflict of interest. The editors and reviewers of this article have no conflicts of interest.

## Figures and Tables

**Table E1:** TRILUMINATE pivotal trial patient eligibility criteria

Inclusion criteria Patients must meet ALL of the following inclusion criteria to participate in the trial: 1. In the judgment of the site local heart team, patient has been adequately treated per applicable standards (including medical management) and stable for at least 30 days as follows: • Optimized medical therapy for treatment of tricuspid regurgitation (TR) (eg, diuretics) • Medical and/or device therapy for mitral regurgitation, atrial fibrillation, coronary artery disease, and heart failure Note: The Eligibility Committee will confirm that the patient has been adequately treated medically. 2. Patient is symptomatic with severe TR despite being optimally treated as described in (1). TR severity is determined by the assessment of a qualifying transthoracic echocardiogram (TTE) and confirmed by the ECL. The ECL also will request a transesophageal echocardiogram (TEE) to confirm TR etiology. Note: If any cardiac procedure(s) occur after eligibility was determined, TR severity will need to be reassessed 30 days after the cardiac procedure(s). 3. The cardiac surgeon of the site local heart team concurs that the patient is at intermediate or greater estimated risk for mortality or morbidity with tricuspid valve surgery. 4. New York Heart Association functional class II or III or ambulatory class IV. 5. In the judgment of the TriClip implanting investigator, femoral vein access is feasible and can accommodate a 25 Fr catheter. 6. Age ≥18 years at the time of consent. 7. Patient must provide written informed consent prior to any trial related procedure. Exclusion criteria Patients who meet any of the following exclusion criteria may NOT participate in the trial: 1. Systolic pulmonary artery pressure >70 mmHg or fixed precapillary pulmonary hypertension as assessed by right heart catheterization. 2. Severe uncontrolled hypertension (systolic blood pressure ≥180 mmHg and/or diastolic blood pressure ≥110 mmHg). 3. Any prior tricuspid valve procedure that would interfere with placement of the TriClip device. 4. Indication for left-sided (eg, severe aortic stenosis, severe mitral regurgitation) or pulmonary valve correction prior 60 days. Note: Patients with concomitant mitral and tricuspid valve disease will have the option of receiving treatment for mitral regurgitation and waiting 60 days prior to being reassessed for the trial. 5. Pacemaker or implantable cardioverter-defibrillator leads that would prevent appropriate placement of the TriClip clip. 6. Tricuspid valve stenosis, defined as a tricuspid valve orifice ≤1.0 cm^2^ and/or mean gradient ≥5 mmHg as measured by the ECL. 7. Left ventricular ejection fraction ≤20%. 8. Tricuspid valve leaflet anatomy that may preclude clip implantation, proper clip positioning on the leaflets or sufficient reduction in TR. This may include: a. Evidence of calcification in the grasping area. b. Presence of a severe coaptation defect (>2 cm) of the tricuspid leaflets. c. Severe leaflet defect(s) preventing proper device placement. d. Ebstein anomaly, identified by a normal annulus position while the valve leaflets are attached to the walls and septum of the right ventricle. 9. Tricuspid valve anatomy not evaluable by TTE and TEE. 10. Active endocarditis or active rheumatic heart disease or leaflets degenerated from rheumatic disease (ie, noncompliant, perforated). 11. Myocardial infarction or known unstable angina within previous 30 days. 12. Percutaneous coronary intervention within previous 30 days. 13. Hemodynamic instability, defined as systolic blood pressure <90 mmHg with or without afterload reduction, cardiogenic shock, or the need for inotropic support or an intra-aortic balloon pump or other hemodynamic support device. 14. Cerebrovascular accident within previous 90 days. 15. Chronic dialysis. 16. Bleeding disorders or hypercoagulable state. 17. Active peptic ulcer or active gastrointestinal bleeding. 18. Contraindication, allergy or hypersensitivity to dual antiplatelet and anticoagulant therapy Note: Contraindication to either antiplatelet or anticoagulant therapy (individually not both therapies) is not an exclusion criterion. 19. Ongoing infection requiring current antibiotic therapy (if temporary illness, patients may enroll 30 days after discontinuation of antibiotics with no active infection). 20. Known allergy or hypersensitivity to device materials. 21. Evidence of intracardiac, inferior vena cava, or femoral venous mass, thrombus or vegetation. 22. Life expectancy of less than 12 months. 23. Patient is currently participating in another clinical trial that has not yet completed its primary endpoint. 24. Patient is currently participating in another clinical investigation for valvular heart disease(s). 25. Pregnant or nursing patients and those who plan pregnancy during the clinical investigation follow-up period. Female patients of child-bearing potential are required to have a negative pregnancy test done within 7 days of the baseline visit per site standard test. Female patients of childbearing potential should be instructed to use safe contraception (eg, intrauterine devices, hormonal contraceptives: contraceptive pills, implants, transdermal patches hormonal vaginal devices, injections with prolonged release.) It is accepted, in certain cases, to include patients having a sterilized regular partner or patients using a double barrier contraceptive method. 26. Presence of other anatomic or comorbid conditions, or other medical, social, or psychological conditions that in the investigator’s opinion could limit the patient’s ability to participate in the clinical investigation or to comply with follow-up requirements or could impact the scientific soundness of the clinical investigation results.

**Table E2:** Echocardiographic evaluation of the heart

Characteristics	Baseline	30 days	1 year	2 years
Patients with prior heart valve surgery (N = 113)				
RA volume, mL	147.9 ± 97.9 (63)	134.3 ± 95.5 (63)	151.8 ± 119.0 (63)	151.4 ± 102.0 (63)
RVEDD-Mid, cm	3.7 ± 0.8 (58)	3.4 ± 0.7 (58)	3.7 ± 0.8 (58)	3.6 ± 0.8 (58)
RVEDD-Base, cm	5.0 ± 0.9 (60)	4.8 ± 1.0 (60)	4.9 ± 0.9 (60)	5.0 ± 0.9 (60)
RV TAPSE, cm	1.6 ± 0.4 (64)	1.5 ± 0.3 (64)	1.4 ± 0.4 (64)	1.3 ± 0.4 (64)
RV FAC, %	37.3 ± 5.0 (51)	37.0 ± 5.7 (51)	38.2 ± 9.5 (51)	35.1 ± 6.5 (51)
TV annulus diameter, end-diastole, cm	4.4 ± 0.9 (62)	4.2 ± 0.8 (62)	4.2 ± 0.7 (62)	4.2 ± 0.8 (62)
Inferior vena cava diameter, cm	2.4 ± 0.7 (63)	2.2 ± 0.7 (63)	2.2 ± 0.6 (63)	2.3 ± 0.5 (63)
RVOT stroke volume, mL	57.6 ± 23.5 (49)	57.4 ± 21.6 (49)	61.0 ± 23.9 (49)	57.0 ± 18.0 (49)
LVEF <40%, % (n/N)	6.5% (4/62)	3.2% (2/62)	6.5% (4/62)	3.2% (2/62)
Patients without prior heart valve surgery (N = 356)				
RA volume, mL	151.5 ± 83.9 (210)	137.9 ± 79.6 (210)	149.6 ± 92.2 (210)	151.6 ± 91.1 (210)
RVEDD-Mid, cm	3.7 ± 0.8 (200)	3.5 ± 0.8 (200)	3.5 ± 0.8 (200)	3.6 ± 0.8 (200)
RVEDD-Base, cm	5.0 ± 0.9 (204)	4.8 ± 0.9 (204)	4.8 ± 0.8 (204)	4.8 ± 0.8 (204)
RV TAPSE, cm	1.7 ± 0.4 (207)	1.6 ± 0.4 (207)	1.6 ± 0.4 (207)	1.4 ± 0.5 (207)
RV FAC, %	37.4 ± 6.6 (185)	34.9 ± 6.7 (185)	36.4 ± 8.2 (186)	34.5 ± 8.0 (186)
TV annulus diameter, end-diastole, cm	4.3 ± 0.8 (210)	4.1 ± 0.8 (210)	4.1 ± 0.8 (210)	4.1 ± 0.7 (210)
Inferior vena cava diameter, cm	2.4 ± 0.8 (201)	2.3 ± 0.7 (201)	2.3 ± 0.7 (201)	2.3 ± 0.6 (201)
RVOT stroke volume, mL	50.8 ± 18.8 (170)	55.6 ± 18.0 (170)	56.8 ± 22.1 (170)	58.5 ± 19.5 (170)
LVEF <40%, % (n/N)	4.2 (8/192)	3.1 (6/192)	4.2 (8/192)	3.1 (6/192)

Values are presented as mean ± SD (n) unless specified otherwise. In this analysis, any follow-up data collected after patients underwent tricuspid valve surgery during the follow-up period were censored.

*RA*, Right atrium; *RVEDD*, right ventricular end-diastolic dimension; *RV*, right ventricular; *TAPSE*, tricuspid annular plane systolic excursion; *FAC*, fractional area change; *TV*, tricuspid valve; *RVOT*, right ventricular outflow tract; *LVEF*, left ventricular outflow tract.
